# Hydrolysed Feather Meal Inclusion in Low Fishmeal Diets for Whiteleg Shrimp (*Penaeus vannamei*)

**DOI:** 10.1155/anu/9967265

**Published:** 2026-02-14

**Authors:** Francesco Bordignon, Luiza Coutinho Costa, Cecília de Souza Valente, Marlise Mauerwerk, Luisa Helena Cazarolli, Caio Henrique do Nascimento Ferreira, Wilson Rogério Boscolo, Eduardo Luis Cupertino Ballester

**Affiliations:** ^1^ Department of Agronomy, Food, Natural resources, Animals and Environment (DAFNAE), University of Padova, Viale dell’Università 16, I-35020, Legnaro, Padova, Italy, unipd.it; ^2^ Shrimp Culture Laboratory, Post Graduation Program in Aquaculture and Sustainable Development, Federal University of Paraná, Palotina, 85953-128, Parana, Brazil, ufpr.br; ^3^ School of Biological and Chemical Sciences, University of Galway, H91TK33, Galway, Ireland, universityofgalway.ie; ^4^ Biochemistry and Genetics Laboratory, Federal University of Fronteira Sul—Laranjeiras do Sul Campus, BR 158, Km 405, Rural Zone, Laranjeiras do Sul, 85301-970, Parana, Brazil; ^5^ Universidade Estadual do Oeste do Paraná, Rua da Faculdade, n° 645, Bloco C, Jardim Santa Maria, Toledo, 85903-000, Paraná, Brazil, unioeste.br

**Keywords:** digestive enzymes, enzymatic hydrolysis, oxidative stress, Pacific whiteleg shrimp, poultry by-products

## Abstract

The global transition toward low‐fishmeal formulations has intensified the search for sustainable and digestible protein alternatives in shrimp aquaculture. Enzymatically hydrolysed feather meal (HFM) represents a promising high‐protein ingredient with enhanced digestibility and bioactive potential. This study evaluated the effects of graded HFM inclusion (0%–5%) on growth performance, digestive enzyme activity, antioxidant status, and muscle composition of *Penaeus vannamei* juveniles. Five isonitrogenous (40.2 ± 1.9% crude protein) and isolipidic (12.3 ± 1.4% crude lipids) diets were formulated with 0%, 1.25%, 2.5%, 3.75%, and 5.0% HFM, replacing part of the soybean meal while maintaining a constant fishmeal inclusion (6%). A total of 100 shrimp (initial weight 1.2 ± 0.1 g; initial length 4.3 ± 0.3 cm) were randomly distributed into 20 tanks (4 tanks per diet; 20 shrimp per diet) and reared for 50 days under controlled clear‐water conditions. Growth performance, feed efficiency, digestive and antioxidant enzyme activities, and abdominal muscle composition were analysed using one‐way ANOVA and polynomial regressions. Growth and feed conversion ratio were unaffected by HFM inclusion (*p* > 0.05). Lipase, cellulase, and carbohydrate‐digesting enzymes remained stable, while trypsin and chymotrypsin showed a mild increase at 1.25%–2.5% inclusion. Glutathione peroxidase activity tended to increase (*p* = 0.10), whereas reduced glutathione was significantly lower in all HFM‐fed groups (*p* < 0.001). Lipid peroxidation (TBARS) and glutathione reductase remained unchanged. Muscle protein and moisture were unaffected, while ether extract showed a modest but significant increase (*p* < 0.001). In conclusion, enzymatically HFM can be safely incorporated up to 5% in low‐fishmeal diets for *P. vannamei* without impairing growth, digestive function, antioxidant defence, or flesh composition, supporting its potential as a sustainable ingredient for modern shrimp feeds.

## 1. Introduction

The Pacific whiteleg shrimp (*Penaeus vannamei*) is a leading aquaculture species worldwide, with 6.8 million tonnes produced in 2022, exceeding oysters (*Crassostrea* spp.), Nile tilapia, and carp [1]. As shrimp aquaculture continues to expand, the development of innovative nutritional strategies is essential to sustain productivity while reducing environmental impact and dependency on fishmeal as the primary protein source [2].

Fishmeal inclusion in practical diets for *P. vannamei* typically ranges from 12% to 25% [3, 4]. Recent research has sought to further reduce these levels, testing inclusion rates that ranged from 8% to 20% [5–7]. However, such reduction often necessitates supplementation with probiotics or synthetic amino acids to offset potential limitations in digestibility and essential amino acid provision [6, 7]. More recently, fishmeal‐free formulations incorporating high levels of poultry by‐product meal (PBM) have demonstrated promising results [5, 8].

The rising demand for PBM as a fishmeal substitute has led to a notable price increase (USD 900/MT [8]), making it compete with other conventional sources (e.g., soybean meal) or alternative ones (e.g., bovine‐pig by‐products [8]). Among poultry by‐products, feather meal represents another abundant (estimated European annual production: 175,000 tonnes [9]), protein‐rich (>78% crude protein [10]), and relatively cheap (USD 662/MT [11]) raw material. Nevertheless, the keratin‐based structure of conventional feather meal limits its digestibility [12]. Enzymatic hydrolysis offers a viable approach to overcome this limitation, improving the solubility and bioavailability of feather proteins while generating bioactive peptides with potential physiological benefits [13]. In fact, these hydrolysates typically yield peptides of uniform size and favourable structural properties, enhancing digestibility [14] and providing functional advantages such as antioxidant, antimicrobial, and immunomodulatory activities [15, 16].

Despite these advantages, only a few studies have assessed feather meal inclusion in *P. vannamei* diets [10, 14]. High inclusion levels (16–24.5%) have been shown to impair growth performance [10], likely due to limited digestibility and amino acid imbalances. In contrast, moderate inclusions of enzymatically hydrolysed feather meal (HFM; 4.5%–9%), co‐extruded with soybean meal, maintained shrimp growth performance when diets contained 15–18.5% of fishmeal [14]. However, no information is available about the suitability of feather meal inclusion in grow‐out diets with reduced levels (<10%) of fishmeal.

Beyond growth performance, evaluating the physiological responses of shrimp to novel feed ingredients is crucial for assessing their suitability. Digestive enzyme activity reflects nutrient utilisation efficiency, while antioxidant responses indicate the ability to maintain cellular homeostasis under oxidative stress—conditions often associated with high inclusion levels of plant‐based ingredients in low‐fishmeal diets [17]. Bioactive peptides derived from feather hydrolysates may modulate these physiological mechanisms and enhance antioxidant capacity [15, 18].

Therefore, the present study aimed to evaluate the effects of graded inclusion levels (0%–5%) of HFM in low‐fishmeal diets (6%) on growth performance, digestive enzyme activity, antioxidant response, welfare indicators, and whole‐body composition of *P. vannamei*.

## 2. Materials and Methods

### 2.1. Experimental Design and Diet Formulation

The feeding trial lasted 50 days and was conducted at the Shrimp Farming Laboratory, Centre for Research and Development in Sustainable Aquaculture, Federal University of Paraná (UFPR; Maripá, PR, Brazil). A total of 100 mixed‐sex *P. vannamei* (Speed‐line, Aquatec; Canguaretama, Rio Grande do Norte, Brazil) juveniles (initial weight 1.2 ± 0.1 g; initial length 4.3 ± 0.3 cm) were used. Shrimp were randomly distributed into 20 circular experimental units (100 L capacity; 93 L working volume) of a clear‐water recirculation system (flow rate: 1400 L h^−1^) connected to a biological filter (600 L capacity; 500 L working volume). The system was installed in a temperature‐controlled room with a 12:12 h light:dark photoperiod, and each unit was equipped with continuous aeration. Salinity was adjusted to 15 g L^−1^ using a commercial salt mix (Blue Treasure Reef Sea Salt, Qingdao Sea‐Salt Aquarium Technology Co., Ltd., Qingdao, China).

Shrimp were stocked at a density of five individuals per experimental unit. Animals were fed six times daily (03:00, 08:30, 11:00, 14:00, 17:00, and 22:00 h). Feeding rates were calculated based on an estimated minimum growth of 1 g week^−1^ and a maximum feed conversion ratio of 1.5. During the trial, biometric measurements (i.e., body weight, body length and antenna length) were performed at the beginning of the trial and every 7 days thereafter, while rearing units were syphoned daily to remove uneaten feed and organic waste.

The experimental design was completely randomised, consisting of five dietary treatments with graded levels of enzymatically HFM inclusion: 0% (control), 1.25% (diet HFM 1.25), 2.5% (diet HFM 2.5), 3.75% (diet HFM 3.75), and 5.0% (diet HFM 5.0), with four replicates per treatment. The inclusion of HFM was performed by partially replacing soybean meal. To maintain diets to be isonitrogenous (40.2 ± 1.9% crude protein) and isolipidic (12.3 ± 1.4% crude lipids), wheat bran was proportionally increased, and minor adjustments were made to DL‐methionine, L‐lysine, mineral sources, and lipid components. Poultry viscera meal (8%), meat and bone meal (15%), and fish oil were maintained constant across all treatments. Diets were formulated to meet *P. vannamei* nutritional requirements [19]. The HFM (79.6% crude protein; 20.5 MJ/kg gross energy; 0.09% calcium; 0.12% phosphorous, as fed) was supplied by BRF S.A. (Toledo, Paraná, Brazil). The ingredients and proximate composition of the experimental diets are presented in Table 1.

**Table 1:** Ingredient composition (g kg^−1^ diet) and proximate composition of experimental diets for *Penaeus vannamei* during the grow‐out phase containing different levels of hydrolysed feather meal.

Ingredient (g kg^−1^ diet)	Experimental diets				
	Control	HFM 1.25	HFM 2.5	HFM 3.75	HFM 5.0
Soybean meal	350.0	321.8	293.5	265.3	237.0
Wheat bran	102.8	119.3	135.7	152.1	168.5
Poultry viscera meal	80.0	80.0	80.0	80.0	80.0
Fishmeal	61.1	61.1	61.1	61.0	61.0
Meat and bone meal	150.0	150.0	150.0	150.0	150.0
Haemoglobin meal	30.0	30.0	30.0	30.0	30.0
Ground corn	100.0	100.0	100.0	100.0	100.0
Hydrolysed feather meal	0.0	12.5	25.0	37.5	50.0
Antifungal agent	1.0	1.0	1.0	1.0	1.0
Antioxidant	0.2	0.2	0.2	0.2	0.2
Binder	5.0	5.0	5.0	5.0	5.0
Limestone	8.5	8.8	8.9	9.1	9.2
Potassium chloride	6.1	6.7	7.4	8.0	8.6
DL‐methionine	3.6	3.7	3.7	3.8	3.8
Dicalcium phosphate	3.1	3.0	2.9	2.8	2.7
L‐lysine	0.6	0.5	0.9	1.4	2.4
Soy lecithin	20.0	20.0	20.0	20.0	20.0
Fish oil	64.4	63.7	62.9	62.1	61.3
Premix^a^	8.0	8.0	8.0	8.0	8.0
Sodium chloride	5.6	4.6	3.5	2.5	1.4
Proximate composition					
Dry matter, %	8.7 ± 1.0	4.5 ± 0.6	5.2 ± 0.7	6.5 ± 0.9	5.6 ± 0.7
Crude protein, %	39.0 ± 1.9	41.0 ± 1.9	40.9 ± 1.9	40.2 ± 1.9	40.1 ± 1.9
Crude lipids, %	11.9 ± 1.4	12.5 ± 1.4	12.3 ± 1.4	12.3 ± 1.4	12.6 ± 1.4
Crude fibre, %	2.8 ± 0.6	4.7 ± 0.9	5.1 ± 0.9	5.1 ± 0.9	2.9 ± 0.7
Ash, %	13.7 ± 0.9	14.0 ± 0.9	13.7 ± 0.9	13.2 ± 0.4	7.2 ± 0.7

*Note:* 1,000,000 IU; vitamin D_3_, 500,000 IU; vitamin E, 20,000 mg; vitamin K_3_, 500 mg; vitamin B_1_, 1900 mg; vitamin B_2_, 2000 mg; vitamin B_6_, 2400 mg; vitamin B_12_, 3500 mg; folic acid, 200 mg; calcium pantothenate, 4000 mg; vitamin C, 25 g; biotin, 40 mg; niacin, 5000 mg; Fe, 12.5 g; Cu, 2000 mg; Mn, 7500 mg; Zn, 25 g; I200 mg; Se, 70 mg.

^a^Vitamin‐mineral premix supplied per kg of diet: vitamin A.

### 2.2. Water Quality

Water quality was monitored throughout the trial. Temperature (27.6 ± 1.21°C), dissolved oxygen (6.78 ± 0.41 mg L^−1^), and pH (7.67 ± 0.15) were measured daily using a multiparameter probe (Hanna HI98196, Hanna Instruments, Woonsocket, Rhode Island, USA). Salinity (15.0 ± 0.0 g L^−1^) was checked weekly using a manual refractometer (Biobrix model 211; Atago, Atago Co., Ltd., Tokyo, Japan). Alkalinity (1278 ± 21 mg L^−1^ CaCO_3_) and hardness (2043 ± 57 mg L^−1^ CaCO_3_) were measured every 2 weeks, while total ammonia nitrogen (0.02 ± 0.02 mg L^−1^) and nitrite (0.08 ± 0.09 mg L^−1^) were determined three times per week following APHA [20] protocols. All parameters remained within recommended ranges for *P. vannamei* culture [21].

### 2.3. Zootechnical Performance and Welfare Assessment

At the end of the trial, shrimp were weighed and measured for total length and antenna length. The following indicators were also calculated:

• Survival (%) = (final number of shrimp/initial number of shrimp) × 100
• Feed conversion ratio (FCR) = g feed supplied/g biomass gain

Animal welfare was assessed using total antenna length as a morphological indicator [22].

### 2.4. Digestive Enzyme Activity and Antioxidant Status

At the end of the trial, hepatopancreases were collected from three shrimp per tank (12 shrimp per diet), immediately frozen in liquid nitrogen, and transported to the Biochemistry and Genetics Laboratory, Federal University of Southern Frontier (Laranjeiras do Sul, PR). Samples were homogenised in 8% saline using a homogeniser (IKA T10 basic) and centrifuged at 12,000×*g* for 10 min at 4°C (Sigma 3–16 KL). The supernatant was used for analyses.

Total soluble protein was determined by the Bradford method [23]. Digestive enzyme activities were analysed as follows: cellulase, maltase, sucrase, and lipase [24]; trypsin and chymotrypsin [25]. Antioxidant system parameters included glutathione reductase (GR, [26]), glutathione S‐transferase (GST, [27]), and glutathione peroxidase (GPx, [28]), all based on NADPH oxidation. Reduced glutathione (GSH) was measured following Sedlak and Lindsay [29] and expressed as µM mg^−1^ protein. Lipid peroxidation (TBARS) was quantified spectrophotometrically at 535 nm.

### 2.5. Chemical Analyses

Proximate composition analyses (moisture, crude protein, ether extract, ash) were performed on the abdominal muscle of five shrimp per tank (20 shrimp per diet) and on the experimental diets, following AOAC [30]. Moisture was determined by oven‐drying at 105°C for 12 h, and ash by incineration at 600°C for 3 h. Crude protein was analysed using the Kjeldahl method, and ether extract by petroleum ether extraction (ANKOM XT10 system). All analyses were performed in triplicate at the Animal Feed and Nutrition Laboratory (LANA/UFPR, Brazil).

### 2.6. Statistical Analysis

Data were first tested for normality and homoscedasticity. Differences among treatments were then assessed by one‐way analysis of variance (ANOVA), with diet as the main effect. When significant effects were found, means were compared by Tukey’s post‐hoc test at a 5% significance level (α = 0.05). In addition, polynomial (quadratic) regression analyses were conducted to explore dose–response trends among treatments. All statistical analyses were performed in R (version 4.5.1) using RStudio IDE (version 2025.09.1‐401).

## 3. Results and Discussion

### 3.1. Animal Performance and Welfare Assessment

Dietary HFM inclusion significantly affected shrimp total length (*p* < 0.001), with slightly higher values observed in shrimp fed the control and 1.25% HFM diets compared with the other treatments (Figure 1). However, the magnitude of these differences was modest and not reflected in other growth indicators. Final body weight (10.5 ± 1.7 g), antenna length (12.3 ± 3.9 cm), biomass gain (49.0 ± 5.4 g), FCR (1.15 ± 0.14), and survival (91 ± 15%; data not shown) did not differ significantly among experimental groups. The absence of negative effects on performance demonstrates that moderate inclusion of HFM (up to 5%) can be safely incorporated into *P. vannamei* grow‐out diets containing only 6% fishmeal. This finding is particularly relevant given the global trend toward reduced fishmeal use in aquafeeds [2]. Earlier studies reported performance impairment at high inclusion levels of unprocessed feather meal (16%–24.5%) due to poor digestibility and amino acid imbalance [10]. In contrast, the present results suggest that enzymatic hydrolysis enhances the nutritional quality and digestibility of feather proteins, allowing their inclusion in low‐fishmeal formulations without compromising shrimp growth. This aligns with the findings of Mendoza et al. [14], who observed comparable performance when HFM (4.5%–9%) was included in diets containing higher fishmeal levels (15%–18.5%). Based on the current outcomes, testing higher HFM inclusion levels of HFM could be valuable; however, excessive inclusion (>10%) may be impractical due to the lower amino acid digestibility of feather meal compared with other poultry by‐product ingredients [31]; [32].

Figure 1Performance (mean ± SD) of Pacific whiteleg shrimp (*Penaeus vannamei*) fed experimental diets containing increasing levels (0%–5%) of enzymatically hydrolysed feather meal (HFM) during a 50‐day feeding trial. Panels show (a) final body weight, (b) total length, (c) antenna length, and (d) feed conversion ratio. Data are presented as boxplots with individual observations (a, b, c) and tank values (d). Different superscript letters indicate significant differences among dietary treatments (*p* < 0.05).

**Figure:**
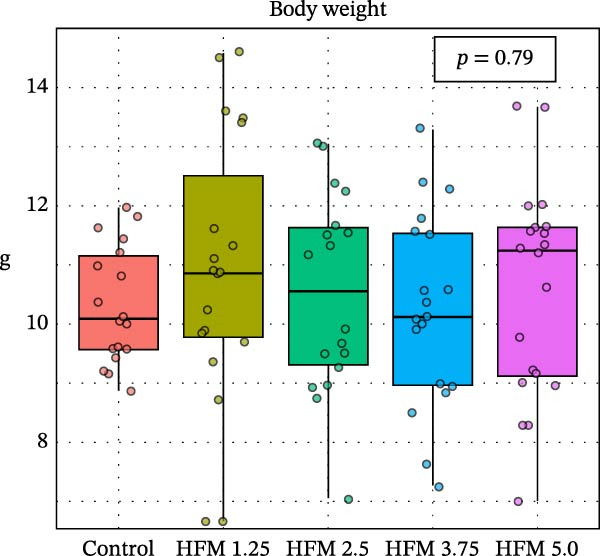
(a)

**Figure:**
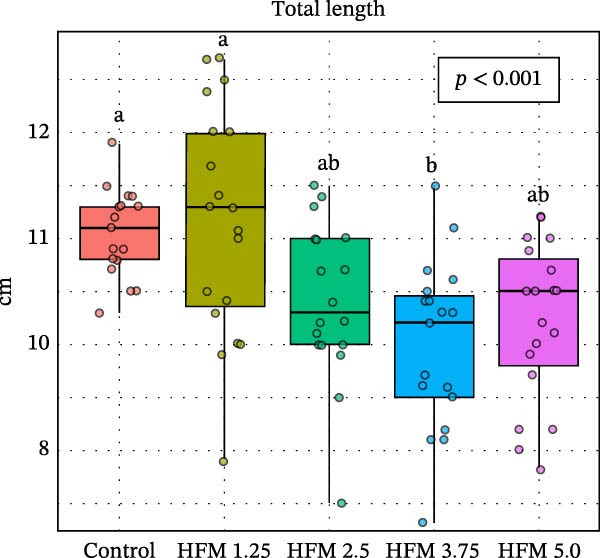
(b)

**Figure:**
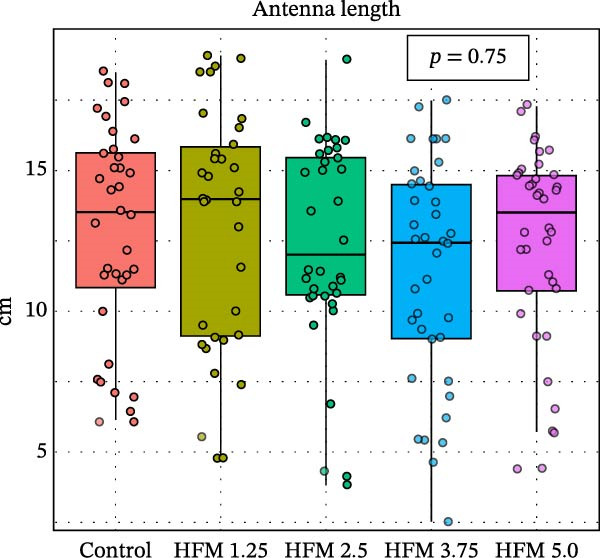
(c)

**Figure:**
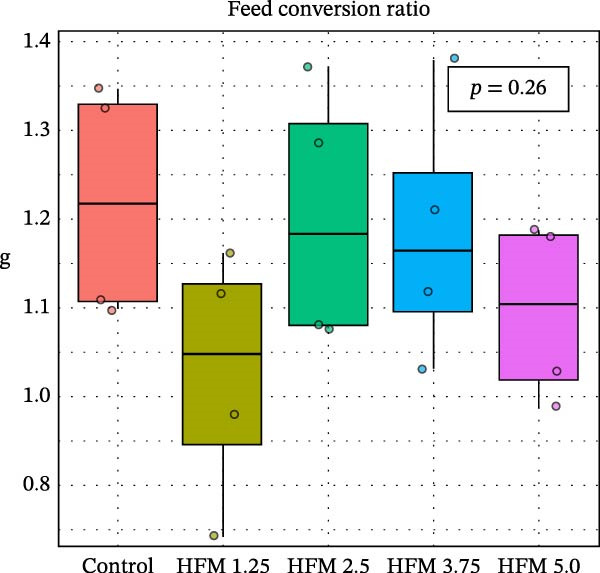
(d)

Our results show that *P. vannamei* can maintain satisfactory growth and survival when fed diets containing only 6% fishmeal, highlighting the feasibility of formulating nutritionally balanced feeds with minimal reliance on marine ingredients. Across studies, *P. vannamei* juveniles maintained growth performance under diets containing low or even zero levels of fishmeal, provided that the overall nutrient profile is properly balanced. In clear‐water systems, shrimp fed diets containing 8%–12% fishmeal achieved comparable final weight and feed efficiency to those receiving higher marine protein levels, while complete replacement of fishmeal with alternative animal or plant proteins only slightly affected performance under biofloc conditions [5]. In diets where fishmeal inclusion was reduced from 20% to 10% and replaced by soybean and PBMs, shrimp growth and feed conversion remained unaffected, confirming that fishmeal levels as low as 10% can sustain optimal performance when amino acid balance and ingredient digestibility are adequately managed [6, 7]. In fishmeal‐free diets, where PBM was substituted with 13.6%–40.7% bovine by‐product meal, shrimp also exhibited good growth performance and feed efficiency, although a slight reduction in survival was observed at the highest inclusion levels [8]. Comparable results were reported when fishmeal inclusion was reduced from 30% to 15% or completely removed and replaced with mixtures of poultry by‐product, krill, and corn gluten meals, with no adverse effects on growth performance [33]. Similarly, when fishmeal levels were lowered from 20% to 7%, methionine supplementation (0.15% Met–Met) fully restored growth and feed efficiency, highlighting the importance of precise amino acid balancing in low‐fishmeal formulations [17].

Antennal integrity has been identified as a sensitive morphological indicator of shrimp welfare, reflecting environmental adequacy and absence of chronic stress [22]. The maintenance of antenna length across all diets suggests that HFM inclusion did not compromise welfare (Table 2), supporting its safety as an alternative ingredient.

**Table 2:** Performance (mean ± SD) of Pacific whiteleg shrimp (*Penaeus vannamei*) fed experimental diets containing increasing levels (0%–5%) of enzymatically hydrolysed feather meal (HFM) during a 50‐day feeding trial.

Variable	Experimental diets					*p*‐Value
	Control	HFM 1.25	HFM 2.5	HFM 3.75	HFM 5.0	
Final weight (g)	10.3 ± 1.0	10.9 ± 2.3	10.5 ± 1.7	10.2 ± 1.7	10.6 ± 1.8	*0.79*
Total length (cm)	11.1 ± 0.8^a^	11.2 ± 1.2^a^	10.4 ± 0.6^ab^	9.8 ± 0.7^b^	10.2 ± 0.8^ab^	*<0.001*
Antenna length (cm)	10.7 ± 0.8	11.1 ± 1.1	10.5 ± 0.6	10.2 ± 0.8	10.2 ± 0.7	*0.75*
Biomass gain (g)	40.6 ± 5.9	46.0 ± 3.7	41.5 ± 6.0	42.8 ± 5.7	45.1 ± 6.0	*0.62*
FCR	1.22 ± 0.14	1.02 ± 0.15	1.20 ± 0.15	1.18 ± 0.15	1.10 ± 0.10	*0.26*

*Note:* Different superscript letters in the same row indicate significant differences among experimental diets.

Abbreviation: FCR, feed conversion ratio.

### 3.2. Digestive Enzyme Activity

No significant differences were detected in the activities of lipase, cellulase, maltase, or sucrase, indicating that HFM inclusion did not affect lipid or carbohydrate digestion (Figure 2). Trypsin and chymotrypsin activities were also unaffected, although a tendency for an increase in trypsin (*p* = 0.06) was observed at moderate inclusion levels (1.25%–3.75%).

Figure 2Activities in digestive enzymes (mean ± SD), including chymotrypsin (a), trypsin (b), maltase (c), sucrase (d), cellulase (e) and lipase (f), of the hepatopancreas of Pacific whiteleg shrimp (*Penaeus vannamei*) fed experimental diets containing increasing levels (0%–5%) of enzymatically hydrolysed feather meal (HFM) during a 50‐day feeding trial.

**Figure:**
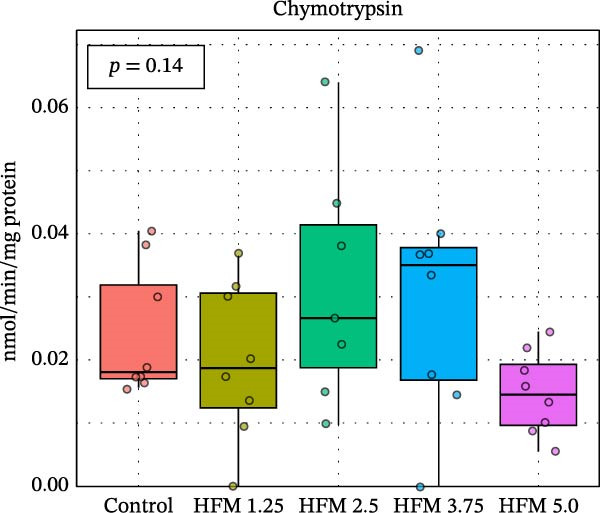
(a)

**Figure:**
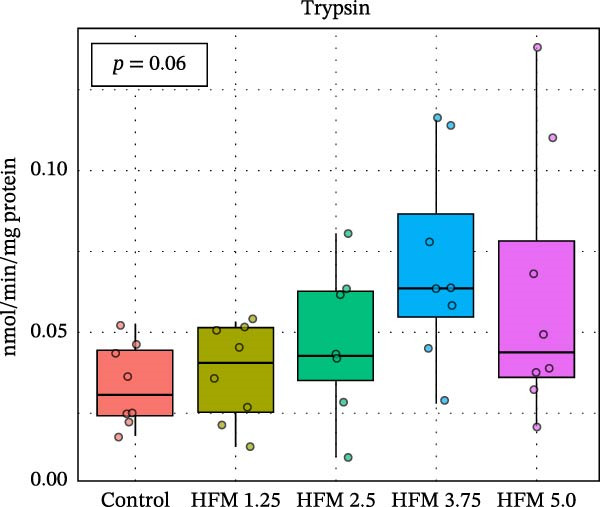
(b)

**Figure:**
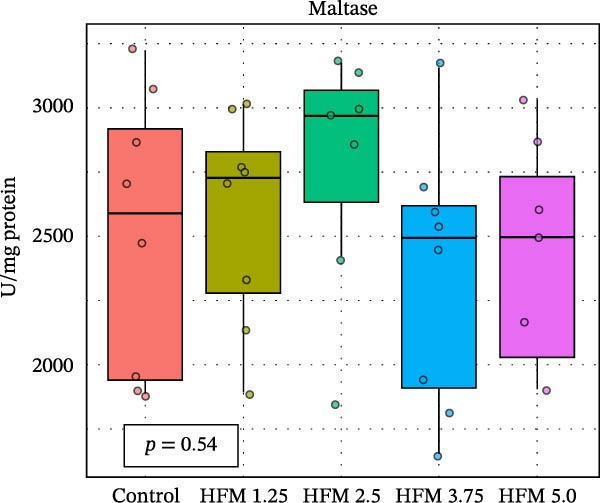
(c)

**Figure:**
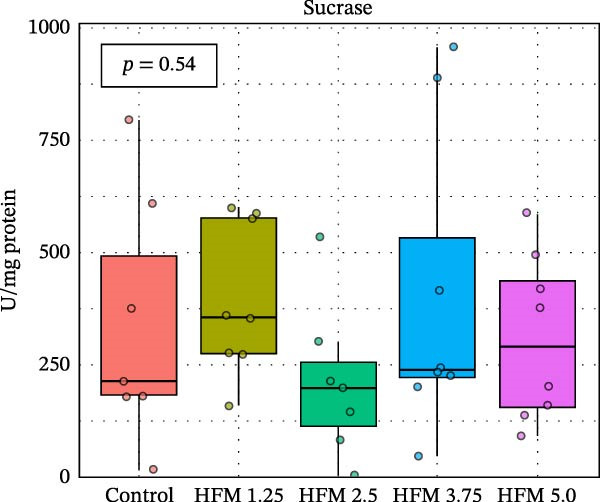
(d)

**Figure:**
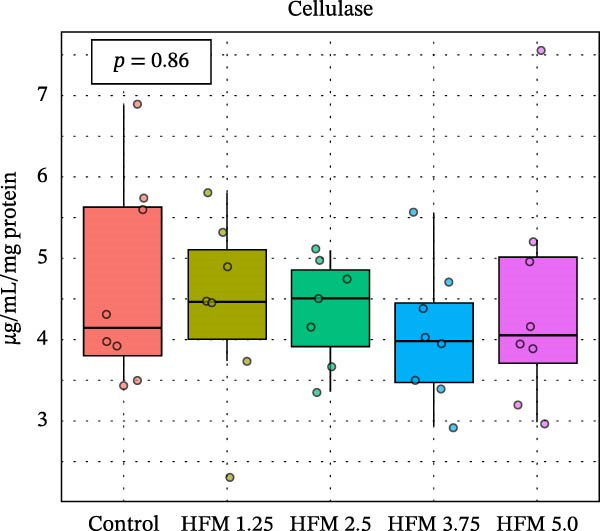
(e)

**Figure:**
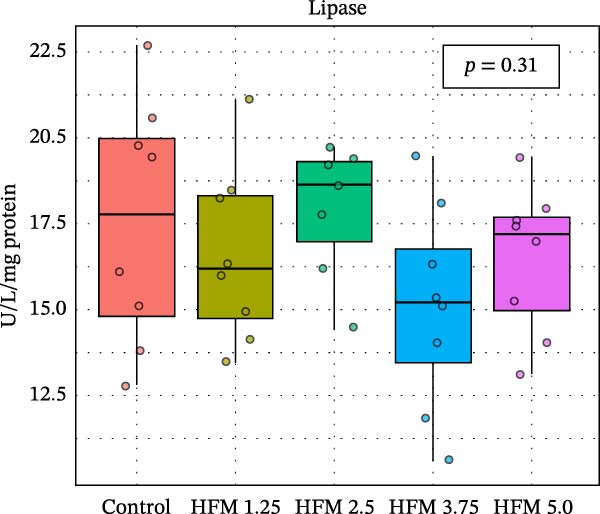
(f)

Comparable enzyme stability has been observed in *P. vannamei* fed low‐fishmeal diets containing properly balanced alternative proteins. Substitution of fishmeal with PBM up to 8% maintained lipase and trypsin activity, whereas higher PBM levels (≥16%) caused a marked decline in both enzymes, along with intestinal wall thinning [34]. Conversely, diets combining PBM, single‐cell protein, insect, and plant ingredients, completely replacing fishmeal, enhanced hepatopancreatic amylase, lipase, and protease activities, likely reflecting complementary nutrient profiles and improved digestibility [35]. Similarly, partial fishmeal replacement (50% replacement; 10% fishmeal inclusion) with soybean and PBM supplemented with methionine and probiotics significantly increased hepatopancreatic amylase, protease, and lipase activities [6], while reduced enzymatic activity is commonly observed in non‐supplemented low‐fishmeal diets [36, 37].

These contrasting patterns across studies indicate that digestive enzyme responses depend more on dietary balance and functional supplementation than on fishmeal inclusion per se. The enzymatic hydrolysis of feather proteins likely enhanced peptide solubility and availability [38], explaining the slight stimulation of proteolytic activity at intermediate HFM levels (Figure 1), whereas the absence of further increases at higher inclusion suggests that enzymatic capacity was already optimised. Moreover, maintaining amino acid balance—particularly sulphur amino acids such as methionine—has been shown to support protease and lipase activities in fish [39, 40], while probiotics can further stimulate hepatopancreatic enzyme secretion and nutrient absorption [6]. Collectively, these findings confirm that moderate incorporation of HFM into low‐fishmeal diets provides a digestible protein source without compromising digestive efficiency or enzyme function.

### 3.3. Antioxidant Response

As for the antioxidant enzyme activities and oxidative stress, only GSH showed a significant dietary effect (*p* < 0.001) (Figure 3). Shrimp fed the control diet exhibited higher GSH concentrations (13.70 ± 2.63 µmol g^−1^ protein) than all HFM‐fed groups (7.50–9.21 µmol g^−1^ protein), indicating increased utilisation of glutathione in oxidative detoxification. GPx activity tended to rise with HFM inclusion (*p* = 0.10), reaching its highest value at 1.25% HFM (0.80 ± 0.23 U mg^−1^ protein), whereas GST (0.30–0.51 U mg^−1^ protein), GR (0.18–0.28 U mg^−1^ protein), and TBARS (0.44 ± 0.13 nmol MDA mg^−1^ protein) remained unchanged (*p* > 0.05) (Figure 3). The decrease in GSH coupled with a modest rise in GPx activity suggests an adaptive enzymatic response—enhancing peroxide neutralisation while maintaining redox equilibrium. This pattern reflects an efficient antioxidant turnover rather than oxidative stress induction.

Figure 3Antioxidant status (mean ± SD), including thiobarbituric acid reactive substances (TBARS; a), reduced glutathione (GSH; b), glutathione S‐transferase (GST; c), glutathione peroxidase (GPx; d) and glutathione reductase (GR; e), in hepatopancreas of Pacific whiteleg shrimp (*Penaeus vannamei*) fed experimental diets containing increasing levels (0%–5%) of enzymatically hydrolysed feather meal (HFM) during a 50‐day feeding trial. Different superscript letters indicate significant differences among dietary treatments (*p* < 0.05).

**Figure:**
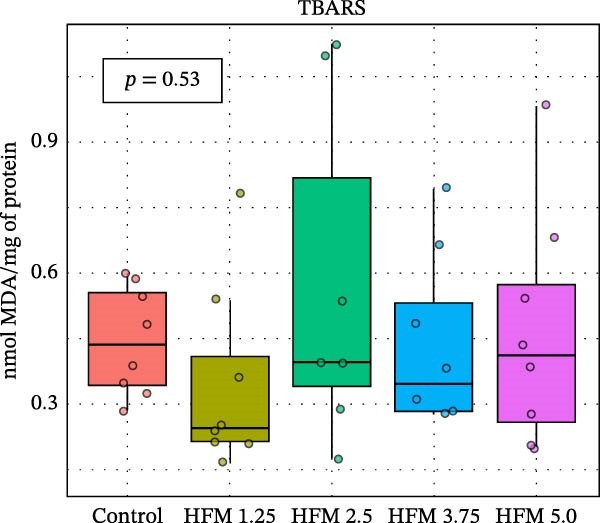
(a)

**Figure:**
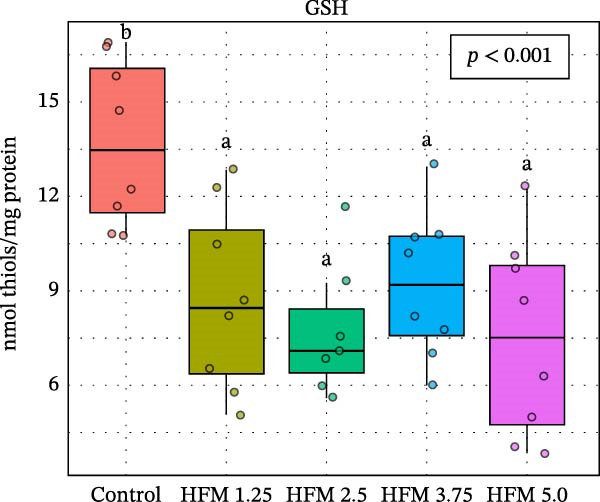
(b)

**Figure:**
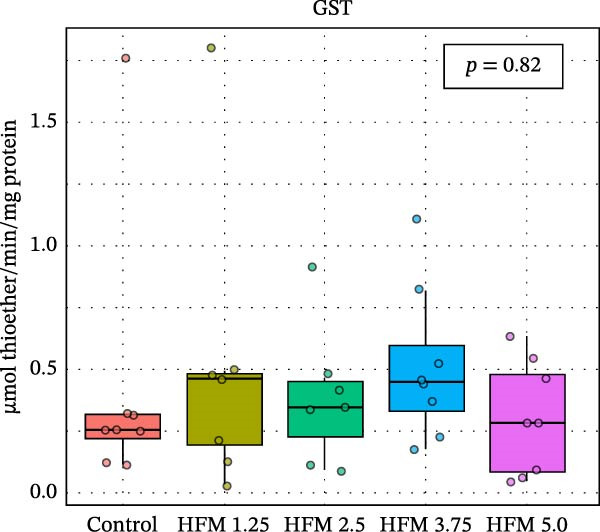
(c)

**Figure:**
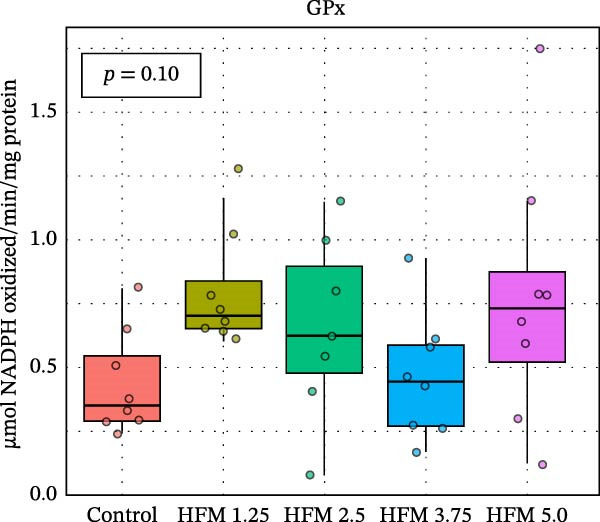
(d)

**Figure:**
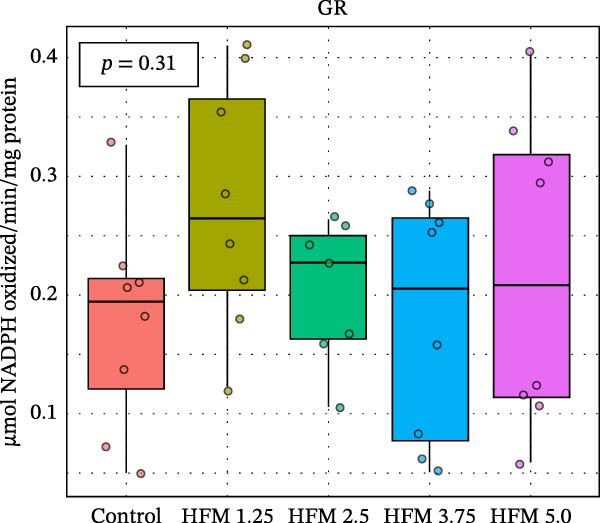
(e)

Comparable responses have been reported in shrimp fed low‐fishmeal diets supplemented with functional amino acids, where methionyl–methionine restored total antioxidant capacity and reduced TBARS at 7% fishmeal inclusion [17]. In contrast, excessive substitution of fishmeal with PBM (≥33%) resulted in suppressed catalase and total antioxidant capacity alongside increased MDA levels, indicative of oxidative stress [34]. Similarly, shrimp fed mixed animal–plant protein diets maintained or enhanced antioxidant gene expression, whereas fully plant‐based formulations led to downregulation of GPx and GST transcripts [33].

### 3.4. Muscle Composition of Shrimp

No significant differences were detected in abdominal muscle dry matter (23.7 ± 0.4%, on average) or crude protein (82.7 ± 0.8% on a dry matter basis) among dietary treatments (*p* > 0.05; Table 3), indicating that the inclusion of up to 5% HFM did not affect protein deposition or water balance. On the other hand, lipid content was higher in shrimp fed HFM 2.5, HFM 3.75, and HFM 5.0 compared with the control (*p* < 0.001), while ash content was higher in HFM 5.0 than in the control and HFM 3.75 (*p* < 0.001).

**Table 3:** Proximate composition (mean ± SD) of abdominal muscle of Pacific whiteleg shrimp (*Penaeus vannamei*) fed experimental diets containing increasing levels (0%–5%) of enzymatically hydrolysed feather meal (HFM) during a 50‐day feeding trial.

Variable	Experimental diets					*p*‐Value
	Control	HFM 1.25	HFM 2.5	HFM 3.75	HFM 5.0	
Dry matter (DM; %)	23.53 ± 0.04	23.54 ± 0.02	23.97 ± 0.70	23.93 ± 0.67	23.35 ± 0.27	*0.63*
Crude protein (%DM)	82.57 ± 0.79	84.04 ± 1.10	82.97 ± 0.02	82.05 ± 0.06	81.93 ± 0.75	*0.15*
Ether extract (%DM)	4.07 ± 0.09^c^	4.30 ± 0.07^bc^	4.41 ± 0.03^abc^	4.75 ± 0.16^a^	4.43 ± 0.04^ab^	*<0.001*
Ash (%DM)	9.70 ± 0.17^b^	10.28 ± 0.26^ab^	10.16 ± 0.02^ab^	9.75 ± 0.30^b^	10.92 ± 0.18^a^	*<0.001*

*Note:* Different superscript letters in the same row indicate significant differences among experimental diets.

Although these differences were statistically significant, the absolute changes were modest (≤0.7% for lipid and ≤1.2% for ash) and are unlikely to represent meaningful physiological or quality alterations. Given that feather meal itself contains negligible fat (<1% ether extract; typically 0.3%–0.8%), the slight increase in lipid likely reflects metabolic adjustments rather than a direct dietary effect. Furthermore, partial replacement of soybean meal with HFM may have slightly altered the energy‐to‐protein ratio, favouring limited lipid deposition without excessive accumulation.

The compositional stability observed across treatments demonstrates that enzymatically HFM can be safely incorporated into low‐fishmeal diets (6% fishmeal) without compromising muscle nutritional quality. Comparable trends have been reported in shrimp fed diets with partial or total replacement of fishmeal using animal or mixed protein sources, where whole‐body protein and lipid contents remained stable [2, 33]. Similarly, low‐fishmeal diets supplemented with methionine maintained muscle protein and balanced lipid metabolism [17], while excessive PBM inclusion (>30%) has been linked to reduced protein and lipid content due to impaired metabolic regulation [34].

## 4. Conclusions

The present study demonstrates that enzymatically HFM can be included up to 5% in low‐fishmeal (6%) diets for *P. vannamei* during the grow‐out phase without impairing growth, feed utilisation, digestive enzyme activity, antioxidant balance, or muscle composition. Overall, enzymatically processed feather meal represents a viable alternative ingredient for sustainable shrimp feed. Its incorporation enables further reduction of fish protein inputs without compromising shrimp performance or product quality, contributing to the development of nutritionally balanced and environmentally responsible aquafeed.

## Author Contributions

**Eduardo Luis Cupertino Ballester**: conceptualisation, resources and funding acquisition, supervision. **Luiza Coutinho Costa**: methodology, original draft preparation. **Cecília de Souza Valente and Francesco Brodignon**: data analysis, writing – review and editing. **Luisa Helena Cazarolli**: digestive enzyme activity and antioxidant status analyses. **Caio Henrique do Nascimento Ferreira**: methodology, statistical analysis. **Marlise Mauerwerk and Wilson Rogério Boscolo**: diet formulation and preparation, chemical analyses.

## Funding

This research was funded by the National Council of Technological and Scientific Development (CNPq), Brazil, under Grant PQ 302500/2025‐1, to Prof. Eduardo Luis Cupertino Ballester.

## Disclosure

All authors have read and agreed to the publishes version of the manuscript. This study was part of Luiza Coutinho Costa Master’s dissertation.

## Ethics Statement

Ethical review and approval were waived for this study due to the current absence of animal protective legislation that includes decapod crustaceans in Brazil (Law Number 11.794, 2008, Brazil). This study did not involve humans or vertebrates.

## Conflicts of Interest

The authors declare no conflicts of interest.

## Data Availability

Data are available upon a reasonable request from the corresponding author.
